# Editorial: Gene regulation of fungal secondary metabolism

**DOI:** 10.3389/fmicb.2023.1260849

**Published:** 2023-08-15

**Authors:** Pinmei Wang, Hee-Soo Park, Wenjie Wang, Wen-Bing Yin

**Affiliations:** ^1^Ocean College, Zhejiang University, Zhoushan, China; ^2^School of Food Science and Biotechnology, Kyungpook National University, Daegu, Republic of Korea; ^3^School of Food Science and Biotechnology, Zhejiang Gongshang University, Hangzhou, China; ^4^State Key Laboratory of Mycology, Institute of Microbiology, Chinese Academy of Sciences, Beijing, China

**Keywords:** gene regulation, transcription factors, fungal secondary metabolism, biosynthesis, secondary metabolites

Fungi are prolific producers of secondary metabolites (SMs), which encompass a wide range of low-molecular-mass compounds. These SMs serve essential functions such as defense against pathogens, inhibition of competing microorganisms, and intercellular communication (Keller, 2019; Yu et al., 2023). Therefore, SMs usually possess dual roles, hazardous mycotoxins (e.g., aflatoxin and patulin), and valuable pharmaceuticals (e.g., penicillin and lovastatin) (Wang et al., 2021b). Surprisingly, genome mining studies have unveiled that the potential of fungi to synthesize SMs has been greatly underestimated, as numerous of biosynthetic gene clusters (BGCs) remain silent or cryptic under laboratory culture conditions (Brakhage, 2013; Wang et al., 2021b). Transcription factors (TFs) play an essential regulatory role in the expression of SM genes (Yin and Keller, 2011; Keller, 2019; Wang et al., 2021a). In recent years, researchers have actively explored the gene regulation mechanism of fungal secondary metabolism by using the methods of genetics, molecular biology and biochemistry (Wang et al., 2022; Zhang et al., 2022; Wei et al., 2023). The aim of this Research Topic is to gain a better understanding of the regulatory elements and network of fungal SMs, providing genetic approaches to manipulate fungal secondary metabolism to produce novel compounds with beneficial properties for medicine, agriculture, and other applications.

This Research Topic contains one review paper and eight original research articles. Deng et al. review article presents a comprehensive summary of perylenequinones (PQs) with literatures spanning from 1967 to 2022. As photosensitizers, PQs have been applied in various fields, including medical, food, agricultural and manufacturing fields. The authors not only collected information of the sources, structure diversity and biological activities of PQs, but also the biosynthetic pathways and regulation mechanism. Besides, strategies are given to enhance PQ production and quality, including modulating regulatory mechanisms, coordinating signal-response pathways, constructing heterologous production platforms, and applying physical and chemical methods to stimulate biosynthesis.

Three research articles investigate the gene regulation of ganoderma triterpenoids (GTs) biosynthesis in *Ganoderma lucidum* and *Ganoderma lingzhi*, both of which are traditional Chinese medicines. GTs exhibit a variety of biological activities, and their biosynthesis is regulated through a complex interplay between environmental and genetic factors. Meng et al. employed a comprehensive approach involving transcriptome and metabolome analyses to investigate the transcription factors (TFs) involved in ganoderic acid (GA) biosynthesis in different developmental stages of *G. lucidum*. By comparing gene expression patterns, they identified that homeobox transcription factor and velvet family protein were responsible for GA biosynthesis, and provided a model for the involvement of TFs in GA biosynthesis during fruiting body formation. The research article by Luo et al. describes an essential function of the well-known methyltransferase LaeA in the regulation of GA in basidiomycete fungus *G. lingzhi*. The results of *laeA* gene deletion and overexpression suggested that LaeA could regulate the expression of GA biosynthetic genes and asexual sporulation. The study by Xu et al. focused on understanding how *G. lucidum* responds to methyl jasmonate, an important elicitor in inducing the production of triterpenes and the biomass of mycelia. Transcriptomic analysis allowed researchers to identify positive and negative transcription factors of GTs that response to methyl jasmonate, and a negative regulator gene *Glmhr1* was found. The study provides new insights into the molecular mechanisms underlying the response of *G. lucidum* to fungal hormones and may facilitate the development of new strategies for enhancing the production of bioactive compounds.

Other two studies explored the role of BcLAE1 and Bcfrp1 in the regulation of abscisic acid (ABA) biosynthesis and growth of *Botrytis cinerea*. The study conducted by Wei et al. demonstrated the significant role of the methyltransferase BcLAE1 in epigenetic regulation of ABA biosynthesis in *B. cinerea*. Chen et al. elucidated the positive role and molecular mechanism of an F-box protein Bcfrp1 in regulating ABA biosynthesis and fungal growth. These studies provide valuable insights into the molecular mechanisms involving the regulation of ABA biosynthesis in *B. cinerea*.

Additionally, two separate investigations have provided knowledge about the mechanisms governing microbe-microbe interactions and plant-microbe interactions, respectively. Brault et al. conducted a study revealing the crucial role of Sib proteins (Sib1, Sib2, and Sib3) in facilitating iron acquisition and cross-feeding interaction between *Schizosaccharomyces pombe* and *Saccharomyces cerevisiae*. These proteins are responsible for the transport of ferrichrome, and their knockout disrupted the transport process and hindered cross-feeding. In another article by Wu et al., they investigated the production and distribution of flavonoids by the endophytic fungus *Aspergillus* sp. Gbtc 2, which was isolated from the root of *Ginkgo biloba*. Through LC-MS analysis, they identified flavonoid metabolites and discovered a unique distribution pattern of these compounds, both intracellularly and extracellularly, within the fungus Gbtc 2. This research expands our understanding of the potential application of endophytic fungi in the industrial flavonoid production. In the study conducted by Wang et al., genomic alterations in *Fusarium proliferatum* strains were examined to understand their role in adaptation and fumonisin production. Through their analysis, a total of 121 distinct genomic loci were identified, implicating 85 potential genes involved in adaptation to diverse environments and fumonisin B1 (FB1) production. Notably, five candidate genes were identified as being closely associated with FB1 production.

The articles within this Research Topic collectively highlight the significance of gene regulation in the biosynthesis of secondary metabolites in fungi. By investigating the regulatory mechanisms governing these processes, researchers can attain a comprehensive comprehension of the molecular mechanisms underlying fungal secondary metabolism. Moreover, these studies enable the identification of potential targets for genetic engineering, aiming to augment the production of bioactive compounds by these organisms. Such findings carry significant implications across various fields including medicine, agriculture, and biotechnology, as they contribute to the exploration and development of novel bioactive compounds of interest.

## Author contributions

PW: Writing—original draft. H-SP: Writing—review and editing. WW: Writing—review and editing. W-BY: Writing—review and editing.
